# Annotations

**Published:** 1900-10-13

**Authors:** 


### Annotations.

### The Question of Chinese Labour in England.

When we hear it seriously proposed to introduce Chinese labour into England with the object of doing what it is stated English labour has failed to do satisfactorily—namely, to wash clothes—many considerations of an economic, sociological, and even ethical nature are raised, in regard to which we will not say more than this, that it will not speak well for the gallantry of the organised trades of this country if, notwithstanding their known readiness to strike for trivial causes in all that concerns men, they allow such a wholesale disturbance of an essentially feminine industry as is threatened by the proposed introduction of Chinese washermen to take place without the strongest protest.

In regard to the sanitary side of the matter, however, a word of warning must be spoken. We hear much about the superiority of the washing done by Chinamen as compared with that done by the machines of the steam laundries and the labour of the English washerwoman, but we take leave to doubt whether this is really the root of the matter. It may be granted, perhaps, that in the washing of clothes hand labour is the best; but probably the real commercial reason for the proposed importation of Chinese is to be found in the fact that their hand labour is the cheapest, and, as sanitarians, we ask at once, Why is the labour of the Chinese the cheapest? We fear that one great reason is to be found in their willingness to accept a lower standard of comfort and sanitation than is recognised as permissible in this country.

There are many objections, some perhaps sentimental, but others very real indeed, to the introduction of the black and yellow races into England. Experience in other countries has amply shown that if an admixture of blood takes place, it only tends to the degradation of the Aryan race, while, if such admixture do not take place, the implantation of such people in our midst, as separate races, breeding among themselves and living apart, is a constant cause of political difficulties.

In view, then, of the fact that the "yellow peril," the danger, that is, of English labour being competed with in its own home by Chinamen and other strangers, although not as yet an urgent matter, is a distinct nuisance and a thing to be guarded against in every way, we think that the most stringent precautions should be taken to prevent the establishment of anything like a Chinatown either in London or anywhere else. We have lately heard many complaints of the difficulty of dealing with the plague in San Francisco, and it now appears that the disease has been dribbling on for the last six months among the Chinese inhabitants; but this is only one example of the complexity of the problems with which the sanitary authorities have for years back been confronted in dealing with these extremely clannish people.

English people always show great reluctance to allow any change to be made in England's position as the home of free trade and as the great open asylum for aliens of all races. But if the free importation of aliens is to be permitted, and if English workers are to be exposed to the open competition of all and sundry who by their own efforts or by the aid of capitalists can manage to be dumped upon our shores, the English people may at least demand that such aliens shall be forced to conform in every respect with the sanitary laws of this country; and it must be confessed that experience shows that this is a very difficult thing to do. Countries which have already suffered from this yellow incursion are practically unanimous in declaring that, useful as the Chinaman may be in certain industries, it is next to impossible to interfere with his home life or to enforce a standard of sanitation such as we demand from his white competitor.

### The Over-examined Medical Student.

In several of the introductory addresses the question of the excessive number of examinations to which the medical student of to-day is subjected became a theme for comment.

Sir William MacCormac in distributing the prizes at St. Thomas's Hospital said that examinations were by no means an unmixed blessing—a sentiment in which the students present appeared heartily to concur. They tended, he said, to specialise the student's work too much; they were hardly a reliable test of his abilities; and there were certainly too many of them.

At St. George's Hospital Dr. Penrose, in alluding to the reconstitution of the University of London, said that he hoped that one good result would be a reduction in the number of examinations which the future student would have to pass in order to obtain his qualifications and degree. He thought that an entrance, an intermediate, and a final examination would be quite sufficient to satisfy all requirements. In this he surely is right. The difficulty, however, is not merely in the number of examinations for any one qualification, but in the fact that the university student has to go up two separate ladders at the same time—one for his degree and the other for his colleges.

### Free Trade in Lectures.

The lament of the medical student is not confined, however, to the matter of his examinations. He is quite as unhappy about his teaching, and on every hand we hear complaints about the miserable waste of time involved in sitting out the lectures, attendance at which is obligatory upon every unfortunate student, however well he may know his work. Dr. Poore, speaking at University College recently, said that some lectures which he had attended in his student days were "mere incoherent and incomprehensible drivel"; and no doubt that is the case with many a student at the present day. Still, as those who sit under Dr. Poore know full well, there are lecturers who can keep their audience, and whose class would always be full even were there no compulsion in the matter. Such men are able by the spoken word to give broad views and to create in their auditors an enthusiasm which they would never have gained by isolated study or by any amount of poring over ponderous tomes. It is in the vitality of the lecturer that his utility lies, and the melancholy truth stares the unfortunate student in the face, that the average lecture is a dull and formal institution out of which but little inspiration can be got. Of the various modes suggested for remedying this wasteful and distressing condition of affairs the two of most interest are the elective system as lately advocated in America, and free trade in lecturing. It has been held that in a good medical school there ought to be extended arrangements for instruction far beyond the limits of any one student's capacity, and that it should to a large extent be left to the student to fill up his brain with the sort of instruction which he finds most helpful. This is the elective system.

We need hardly say that it is hopeless to think of such a system being introduced in England (even if it were desirable), for the student is here tied at every step to curriculum laid down for his guidance. Free trade in lecturing could, however, be introduced at once by any school, and it can hardly be doubted that if it were open to anyone who could show that he was possessed of the necessary "plant" to lecture upon any subject, and if all such discourses were accepted and signed for by the authorities, brilliant lecturers would soon arise, and competition would soon empty the benches of those from whose teaching students feel that they gain no benefit. The medical student of to-day is still "a very much lectured individual," and it is to be hoped that some time in the future his lot may be lightened in this respect. In the meantime the schools ought to ensure that their compulsory lectures are of good quality, and probably this will best be done by the introduction of some degree of free trade in lecturing. Professors are, after all, quite as human as grocers, and will be quite as ready to provide a better article under the stimulus of competition. Unfortunately the system of "composition fees" which prevails in London, according to which the students get their lectures on each subject "at a reduction" on condition of getting all the others at the same shop, tends greatly to conservatism, and leaves the student at the mercy of the "text-book lecturer."

### Dog-ears and Thumb-licking.

At the annual congress of the Library Association recently held in Bristol, a paper was read by Mr. MacAlister, Librarian to the Royal Medical and Chirurgical Society, and Dr. Savage, Bacteriologist to the Cardiff Public Library, on the risk of infection being carried by books from public libraries. On the whole, they did not seem to consider the danger at all great, for they pointed out that books used by patients with diphtheria, enteric fever, and tuberculosis had been examined bacteriologically, with results showing that it appeared next to impossible to convey infection by this means. There was, however, an exception to the general benediction thus given to library books, for when people licked their thumbs to assist in turning over the leaves it would obviously be possible for infection to be carried in a very direct manner from one thumb-licker to another. This, however, is theoretical, and we may doubt whether in any case infection has been proved to have been transmitted in this manner. The condition of the books in some of our public libraries would indeed suggest that thumb-licking must be a very innocent practice, for if it were true that infection were at all frequently transmitted in this way, the thumb-lickers—a tribe afflicted with a peculiarity entailing such risks upon them—ought according to the law of the survival of the fittest to die out quickly. Our public-library books, however, give good evidence that the tribe still thrives, so the danger cannot be inordinately great. At any rate, just as it takes two to make a quarrel, so it ought to take two to transmit a disease by thumb-licking. If, then, we abstain from that easy way of turning pages we may read our library books without alarm.

### Curtains and Mosquitoes.

Among the various experiments which have recently been made as to the transmission of malaria by mosquitoes an interesting point has come out in regard to the suggestion that the number of bites may have something to do with the intensity of the resulting disease. It is no doubt quite possible that a single bite of a sufficiently infected mosquito might do the mischief, but there is much evidence to show that the larger the number of bites the greater the number of parasites which will be found in the blood at a given date. This is a somewhat important matter, because it has been objected by some that it must be a hopeless task for a man to attempt to protect himself absolutely from the attacks of mosquitoes in countries where these creatures abound in untold numbers. But if one recognises that by no means all mosquitoes are infected, perhaps not one in many thousands, and if one believes, as from analogy with other infections one fairly may, that the healthy body is generally able to deal with a small and limited dose of infection, it is reasonable also to believe that a man may often be able by a careful use of mosquito curtains to obtain protection, even though these appliances may not succeed in keeping out the mosquitoes with that absolute completeness which might theoretically seem necessary. It is this consideration which makes it possible to accept the suggestion that protection can be given by curtains. If a single error in tucking in one's curtains would undo all the trouble one had taken, one had better stay at home.

### Hospitals and Lay Managers.

Commenting on the difficulties which have arisen in consequence of the exclusion of the medical staff from the Board of Management of the National Hospital for the Paralysed and Epileptic in London, the *New York Medical News* greatly ridicules the idea of a medical staff composed of some of the ablest physicians in England being shut out from the management of such a hospital.

It says that the very existence of a hospital implies the willingness of good-hearted people to give their money, and able physicians to give their services, for the same altruistic end—the good of the sick—and that "no one will for a moment deny that the physicians are the power by which this end is attained, and that the lay managers are their assistants and co-operators." It adds that most of the larger and best-managed hospitals in America vest executive power in the medical staff, either in the form of a committee, or a representative, or by the appointment of a medical man as chief executive officer of the hospital. "That eminent physicians who are carrying on advanced scientific treatment should be obliged to beg for a voice in the management of the work of which they are the very impulse seems laughable." The medical men must be free to advise and to co-operate with the directors of the hospital, and they in turn must be sufficiently in touch with the medical men to be able to discuss all medical affairs of the hospital without hurting their feelings.

